# Health Literacy in Adults with Chronic Diseases in the Context of Community Health Nursing: A Scoping Review

**DOI:** 10.3390/nursrep13020072

**Published:** 2023-05-24

**Authors:** Annike Morgane Nock, Sabine Metzing, Ivonne-Nadine Jürgensen, Corinna Petersen-Ewert

**Affiliations:** 1 Department of Nursing and Management, Faculty of Business and Social Science, University of Applied Sciences Hamburg, 20099 Hamburg, Germany; 2 School of Nursing Science, Faculty of Health, Witten/Herdecke University, 58455 Witten, Germany

**Keywords:** health literacy, chronic disease, community health nursing, health literacy strategies, primary healthcare

## Abstract

*Background:* Health literacy was identified as a main determinant in self-care of chronic diseases. This results in responsibilities for health professionals for daily practice. For primary care setting, special requirements arise due to the heterogeneity of communities. The objective of this scoping review was to explore and map the scope of the research material on strategies led by community health nurses to improve health literacy in (patients with) chronic diseases. This review aimed to identify gaps in the literature and existing approaches on promoting health literacy by community nurse practitioners. *Methods:* The following criteria were included for the research: Adults with chronic diseases, health literacy, community health nursing and primary healthcare. All types of searches for studies from 1970 to present were carried out in electronic databases and in a Google and a Google Scholar search. The search procedure is presented in a flow chart. *Results:* From all reviewed studies, nine records were included in the review. Findings with regard to the increase in health literacy in self-management of chronically ill patients were identified. *Conclusion:* Studies focusing on specific demands with regard to the role of community health nurses need to be carried out in depth.

## 1. Introduction

In the midst of the global crises and an epidemiological change, the world is currently experiencing the value of health-related communication in a particular way. The ability to find, understand, navigate, explain and to apply health information intensified in individuals and populations. This can be observed in dealing with acute events, such as the COVID-19 pandemic [1], when rapid awareness of relevant information is important [2]. However, notably, it seems fairly relevant in coping with long-term conditions that require the comprehension of complex information [3]. Studies report higher needs for consumer’s understanding in the context of chronic illness [4,5]. 

With the increased number of options in today’s world, new challenges arise for individuals in order to make well informed decisions for themselves and for their relatives. The worth of personal expertise became more essential; for instance, to identify misinformation due to the increasing quantity of information (“infodemic”) in social and mass media [6]. Hence, the availability of quickly comprehensible information is gaining importance, especially in places where people spend their time, such as social media channels (TikTok^®^). Consequently, the concept of health literacy received quite an attention since the pandemic [2] and the increased burden of chronic disease [7]. For researchers, responsibilities of healthcare organizations to provide information and targeting personal competencies are of growing interest in this context. 

The term health literacy firstly appeared in the 1970s [8]. Literature on the subject was frequently related to the medical field, but rarely to the term “health literacy” itself [9]. In the 1990s, the World Health Organization (WHO) shifted the focus from the illness-associated context towards a health-associated perspective. Since then, various measurement tools were developed to quantify dimensions of health literacy [10], and different definitions emerged [11]. Approaches for health literacy in nursing research increased in the last decade [12]. An often cited and broadly used definition by Sørensen et al. [11] describes health literacy multidimensionally. Accordingly, it characterizes health literacy as the ability and incentive of people to examine, understand, consider, request and apply health information in everyday decisions to enhance their (health-related) quality of life [11]. This multi-layered understanding emphasizes social and environmental factors as preconditional to ensure consumers health literacy besides their personal resources. From this societal perspective, the availability of reliable information is mandatory to accomplish it in order to fulfil its purpose. To give people the opportunity to make their own choices, an access to (critical) health information on equal terms is necessary [2,5]. It is evident that the less favorable the social situation, the worse is the assessed health literacy. 

Gradually, health literacy gained importance as a social health determinant [13,14]. According to the WHO strategies ‘Health for All’ and ‘Health in All Policies’ [15], encountering disparities is a key target of our century. Consequently, a broader consideration of health literacy is establishing itself. For example, in the Healthy People 2030 initiative, the OASH [16] highlighted people’s ability to use health information and make “well-informed” rather than “appropriate” decisions. In this regard, the responsibilities of healthcare organizations and the interdisciplinary collaboration are particularly mentioned [16]. Since primary care, in most cases, is the first contact with a healthcare system for the consumer, a special significance derives on the organizational health literacy competency. This applies especially for people with chronic conditions as they are diagnosed, treated and informed about their condition and the course of disease in primary care services [17,18]. As healthcare professionals are key providers to address information to patients, promoting health literacy is obligatory in their daily practice [19]. They have to be able to identify resources/limits of those people and communities to be entirely health literacy responsible [20]. There are several conditions that might have an influence on how health professionals provide information to patients in a fair way. The importance of a user-friendly communication by physicians as a core competency is self-evident [21]. However, study findings reported that during medical consultation, patients described their need for comprehensible information as unsatisfied [22,23]. Thus, the role of nurses [24] in coping with chronic illness and permanent functional disability appeared evidently. Nurses train patients in health-related skills as part of their routine practice [25]. Health related issues are almost always associated with a need for information and advice, especially for patients with a chronic disease which implies a high demand on their knowledge and decision-making in everyday life [4,26]. It is mostly nurses who advocate for consumers and promote patients’ health literacy in their daily practice, both in primary and clinical care settings. They often share and simplify the information provided by other professionals. 

There are established tools for nurses to respond effectively to patients’ health literacy needs and demands on individual information. For example, communication strategies to explain medical information in easy language or to make sure that the care-setting is a shame-free environment [19]. Accordingly, in their role as multipliers, nurses are encouraged to use health literacy approaches to empower patients as well as communities. Various strategies are needed. For example, to make health information accessible, to identify gaps in understanding, and to communicate in a sensitive way regarding the patients’ socio-cultural needs [12,24]. These can be achieved by encouraging consumers to ask questions, speaking slowly or asking questions to determine the patients understanding [27,28]. In the literature, the teach-back method was described as a commonly used strategy for educating people with chronic diseases [29,30]. In a systematic review, Ha Dinh et al. [30] reported on the effects of the teach-back method on disease-related outcomes. The results showed significant positive effects on disease-related knowledge, self-efficacy and adherence, across people receiving the information by teach-back communication [30].

However, the majority of those described strategies stem from the perspective of the entire professional nursing spectrum. Research about health literacy intervention delivered by community health nurses (CHNs) is scarce, although special requirements arise here due to the diversity of the communities: encounters with diverse groups and individuals mean that CHNs are dealing with multiple needs and preferences in their everyday practice. Accordingly, CHNs play a major role in promoting health literacy in communities with a wide range of responsibilities and peculiarities. With regard to Germany, research on health literacy in patients with chronic diseases and the role of nursing is limited. Certainly, there are findings on health literacy in chronic diseases [3]. Recently, Griese [26] reviewed the state of research on health literacy and chronic disease with geographic reference to Germany. However, the potential of community health nursing to empower patient understanding was rarely discussed from a national perspective so far. Therefore, more research should be conducted on the scope of health literacy strategies for patients with chronic diseases delivered by CHNs. This is especially important, as there is currently a discourse in Germany on the implementation of advanced/community nurse practitioners in the German health care system [31]. 

### Aim

Hence, the interest of this scoping review is to identify literature on health literacy strategies of community health nurses in primary healthcare among patients with chronic disease. The review is embedded in a study, which tested and evaluated a community nurse care-concept for patients with a chronic disease in a major German city [32,33]. Therefore, this review aims to provide a first direction on the state of existing material on this topic produced by academic researchers and practitioners from the field. As health literacy in the context of CHN appears as a comprehensive and heterogeneous field of application and research, we assumed a great complexity of literature sources which were not yet comprehensively reviewed. The intention of the scoping review is to identify reported strategies, in order to obtain an overview of evaluated community health nurse-led approaches on health literacy promotion and to highlight possible research gaps in this field. This review will re-examine the existing evidence to derive more specific questions and need for action for clinical nursing practice. A more precise systematic review of the evidence was not indicated in this first step due to this broadly diversified research subject [34]. In order to identify any material on the topic, we both used scientific, peer reviewed sources as well as unpublished material and gray literature from (non-governmental) organizations and professional societies. We approached our research interest based on the following questions:

– What empirical evidence was published on health literacy among people with chronic diseases, and which of these studies referred to community health nursing?
– Which outcome parameters were reported?
– What was reported about community health nurse-led interventions to promote health literacy?

## 2. Materials and Methods

This scoping review followed the guidelines of the JBI’s approach for scoping reviews, including the recommended methodological steps according to Peters et al. [35]. It adhered to the checklist for Preferred Reporting Items for Systematic Reviews and Meta-Analyses extension for Scoping Reviews (PRISMA-ScR) [36]. A preliminary search did not identify a similar scoping or systematic review on this topic. According to our research interest, we defined the main concepts and inclusion characteristics from our review questions based on the PCC (population, concept, context) framework. From this, we defined our search strategy. Details on the main concepts (PCC) were described in our a priori scoping review protocol, registered online in August 2022 in Open Science Framework (2022). 

### 2.1. Inclusion Criteria

*Participants:* Adults aged 18 years and over with at least one chronic disease. 

*Concept:* The concept of health literacy (including operationalization/measurement tools).

*Context:* The scope of community health nursing-led (or advanced nursing practice or public health nursing) methods used to promote health literacy in a primary (or community) care setting. 

### 2.2. Search Strategy

**Figure 1:**
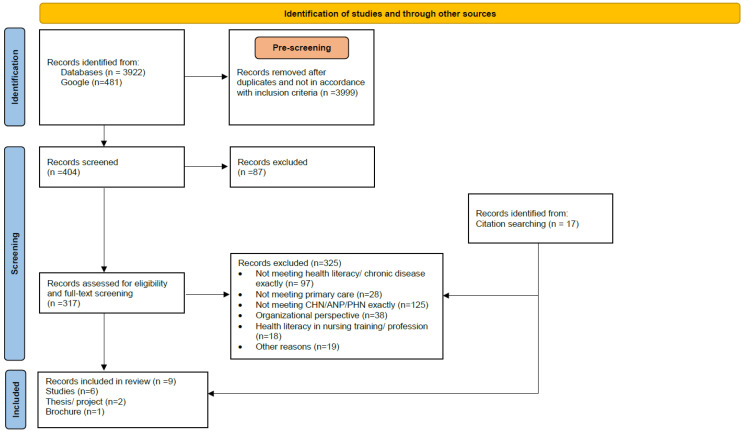
PRISMA Flowchart [37].

Our search procedure and decision-making process in the selection of records is shown in Figure 1 (the PRISMA-ScR Checklist is in the Supplementary Materials) in a PRISMA flowchart [37]. Based on a preliminary search (on 25 March 2022) in MEDLINE (via PubMed), suitable index terms, MeSH headings, keywords and synonyms for health literacy, chronic disease and community health nursing were identified. Subsequently, both reviewers (AMN, INJ) consulted an experienced librarian from the Hamburg University of Applied Sciences on the database selection and search. Thereafter, the search was performed step-by-step (iterative). Additional keywords, synonyms and sources were identified and were subsequently included in order to refine the strategy by each further step. For community health nursing, the related terms “public health nursing”, “advanced practitioner nurse”, “nurse*” and “caregiver*” were used. The search was conducted from August 2022 to January 2023. Firstly, MEDLINE (via PubMed) was searched using MeSH headings for health literacy AND chronic disease, filtered by abstract, free full text, humans and adults aged 18 years and over. The results were mapped according to their reported outcome parameters. The claim to completeness was not the focus, rather refining the search strategy in order to find frequent outcomes reported. In the second step, we added the term community health nursing and related terms into our search path. From all keywords identified, a search syntax was defined, which combined the keywords along with their synonyms using the operators OR/AND. Searches in the electronic databases MEDLINE (Pub-Med), CINAHL, Scopus, CareLit, PsycINFO (via Ovid), LIVIVO, OpenGrey and Web of Science were carried out with no limit to the types of sources and countries, filtering from 1970 to present. Online catalogs of the Hamburg University of Applied Sciences, the German national library, the Swiss national library and the Austrian library network were also screened. The syntax was adapted to the respective options of each single data-base. Additionally, a hand search in Google was conducted to examine websites of topic related organizations, conference reports and other unidentified publications. Frequently mentioned authors on the topic were also scanned. Across all data-bases and Google, the search yielded 4403 results. The search was undertaken by one author (AMN), who screened all results by title/abstract for inclusion criteria and duplicates. To log the search history in detail, the RefHunter protocol template by Hirt and Nordhausen [38] was used. In this step, the second reviewer (I.-N.J.) was only consulted in case of ambiguity. 

### 2.3. Study Selection

In the next step, all rated (*n* = 404) results were uploaded into Citavi 6.10.0.0 (Swiss Academic Software) and remaining duplicates were removed. The records were then assessed for eligibility by both reviewers (AMN and INJ). In this step, the reviewers successively screened titles and abstracts by highlighting the inclusion criteria and sorting the results by category and relevance. If literature was unclear for inclusion, it was categorized as “possibly relevant” or “criteria unclear” and was discussed between the two reviewers. After completing title and abstract screening, *n* = 87 records were excluded and *n* = 317 were determined for full-text assessment. The included full texts were then verified by assessing their content in relation to the main characteristics of health literacy, chronic disease, community health nursing, primary care and synonymous terms. Any ambiguities about eligibility were resolved by further discussion and additional search on terms that were used in the literature. A detailed description of this selection follows in the results section. Furthermore, all reference lists of sources from the full-text review were scanned and *n* = 17 additional sources were identified through this screening. 

### 2.4. Data Extraction

As stated in the protocol, data from publications included in the review were extracted and summarized in a table. They were continuously developed during the review process and included the following details: First author/year/country, study design, participants (sample), methods, type(s) of chronic disease, aim, outcomes, intervention, setting and main findings or recommendations. While one reviewer (AMN) extracted the data, the second reviewer (INJ) cross-checked the information.

## 3. Results

Of the full-text reviewed material, approximately 97% was excluded (*n* = 325) for the following reasons: *N* = 97 results did not exactly meet chronic disease and/or health literacy within their studies. Many studies lacked a clear description of this research topic of interest to our review. During the selection process, we did not find the term community health nursing in many of the papers we read. In the full texts, our pre-determined terms such as community health nursing (CHN), advanced nursing practice (ANP) and public health nursing (PHN) were often not described accurately. Several papers referred to health professionals’ teams or centers. The role of the (employed) nurses was often not clearly defined. In most cases, it was also not clearly described which of the professionals involved exactly planned and implemented the health literacy interventions. Thus, the reviewer tandem frequently shared their individual assessments with each other during the full-text screening and pre-selected the literature material. Each single paper/source from pre-selection was then reviewed independently against this focus. The reviewers discussed a continued agreement for exclusion. Furthermore, one reviewer (AMN) conducted additional internet searches to find the exact designations of uncertain studies to clarify whether inclusion was appropriate (e.g., which degree had the designated nurse). All papers on the topic of health literacy in people with chronic disease that did not exactly refer to the profile CHN, ANP or PHN (*n* = 125) were excluded. A large amount of literature was found concerning health literacy strategies and promotion by different health professions disciplines. The studies reviewed yielded little differentiated perspectives on the specific role of advanced practice nursing in the community in this context. We excluded 40 papers referring to nursing in general, 46 publications on other health professionals, four studies on the role of community health workers and one study on school nurses’ interventions. Furthermore, several of the excluded studies were not conducted in the primary care setting (*n* = 28). Thirty-eight papers examined the perspective of a whole health organization. Health literacy of nursing students was assessed in 18 studies. The remaining material (*n* = 19) was excluded for other reasons. 

### 3.1. Literature on Health Literacy among People with Chronic Disease 

In total, from the reviewed full texts, it can be reported that health literacy was examined across various chronic diseases and patient groups: Type 2 diabetes mellitus, types of cancer, cardiovascular, kidney and respiratory diseases in older adults or indigenous people. E-health literacy among chronically ill patients was also explored in several studies. Looking at the validated instruments used for health literacy measurement, different questionnaires were described in the studies, such as the REALM (Rapid Estimate of Adult Literacy), the HLS-19-COM-P (Communicative Health Literacy in Patient–Physician Communication), the TOFHLA (Test of Functional Health Literacy in Adults) or the HELP Questionnaire (Health Education Literacy in Patients).

From the retrieved full texts, most were scientific studies. However, a large amount of other non-academic publication types—for example, working papers or recommendations for practice on the subject—were also identified. It can be highlighted that several toolkits [39,40,41] on health literacy interventions (such as the teach-back method) were identified in the literature. A total of *n* = 6 video tutorials and reports on health literacy strategies for healthcare professionals and organizations were found. This shows that in addition to scientific studies on this topic, there were also publications on practical recommendations for action. Various nursing associations (such as the American Academy of Nursing) proclaimed a variety of materials and recommendations on their websites. In their policy papers, they called for implementing nurses’ strategies to improve patients’ literacy skills. They advocated for the implementation of nursing-specific health literacy programs and policies [42]. An overview of different evidence-based methods (i.e., teach-back method in all patient communications) was provided by several associations. They can be used as a guidance to support primary care practices in addressing health literacy. However, in this context, increased attention was paid to the group of patients with chronic diseases and to preventive measures to promote health literacy by healthcare professionals or organizations. 

### 3.2. Reported Strategies by Community Health Nurses

From all reviewed full texts (including papers identified from references), we included nine studies for data extraction. They were published between 2016 and 2022. In most of the included papers, health literacy was primarily considered in prevalent chronic diseases. Several of these studies [studies a, b, c, d, h] explored the effects of community nurse-led interventions on different outcomes related to health literacy and chronic disease. Three papers [studies e, f, g, i] reported recommendations for practice and focused on the benefits of CHN/ANP interventions and their role in promoting health literacy in patients (with chronic diseases). The included papers comprised six studies, a master’s thesis and a graduate project found in electronic databases and from reference lists. Additionally, one brochure on tasks and practice of CHNs from Germany was found [study i]. Six papers were written in English and three were written in German. 

Two studies used a quasi-experimental study design (pre-/post intervention) and were conducted in Brazil [study a] and Egypt [study c]. Two studies were brief reports from Austria [study f] and the United States [study e], and one was a study protocol for a randomized controlled trial from China [study b], which was not yet published. Another study from Canada [study d] used a single arm pre-post design and was embedded in an international trial, which was conducted in New Zealand, Australia and Canada. The master’s thesis was a qualitative study from Austria [study g], which explored how community health nurses/advanced counselling nurses can improve health literacy. The thesis was based on expert interviews and included recommendations for practice. The graduate project reported on a quality improvement project (pre-/post study) conducted in Florida, United States [study h]. All of the reported studies are summarized as follows and shown in a short table (Table 1). More details on the results from the included studies are shown in Supplementary Materials.

The majority of the included studies reported on education of chronically ill patients in self-care/self-management of their illness and to increase their (functional) health literacy, self-confidence, self-efficacy and quality of life [studies a, b, c, d, f, h]. Outcomes were examined in patients with diabetes mellitus [studies a, c, h], cardiovascular events and hypertension [studies c, d], cancer diagnosis [study e] or multimorbidity/chronic disease in general [studies b, f, g, i]. Four studies described distinguished health literacy needs of chronically ill patients [studies a, b, d, g]. Three studies focused on disease/medication adherence [studies a, b, c] and patients’ knowledge of medication use [study d]. The data collection methods in the studies ranged from pre- and post- (face-to-face) interviews [studies a, b, c, d] or surveys [study h], expert interviews and a focus group [study g], participant observation [study d] and measurement of changes in physical measures (blood glucose, blood pressure) [study c]. Both quantitative and qualitative methods were applied. The classification of groups/communities studied showed that one study specified the needs of indigenous populations [study d], one focused on a rural community [study h], two studies reported on participants aged over 60 years [studies b, c] and one study on adults in the age range between 30 and 69 years [study a]. An interesting result was that none of the validated instruments on health literacy previously reported in the literature were used in the studies. The interventions implemented in the studies were patient-centered and delivered by advanced/community nurses with further specialization in primary health care settings. The approaches included different methods, such as group or roundtable conversations [studies a, b, c], practical demonstrations [studies a, d], the teach-back method [studies b, e], motivational interviewing [study b], active listening [studies a, b, d], exercising in communication with health professionals [studies a, b, d, h] and feedback [studies b, d, h]. Frequently reported were supporting materials, toolkits [studies a, c, e, g, h, i], as well as online applications [study d] applied during educational sessions. Several of the described interventions aimed to exercise communication skills, to empower the patients for consultations with physicians or other health care professionals.

In terms of the results of the studies, it can be summarized that partial intervention effects were reported with regard to increases in health literacy, self-efficacy, medication knowledge, adherence and participants’ self-confidence [studies a, c, d, h]. 

## 4. Discussion

In this review, the community health nurse’s role in promoting health literacy for patients with chronic disease was explored. In order to enable chronically ill people to participate in their therapy, a high level of health information is required. Patient-centered strategies in primary care contribute to strengthen the health literacy of people in the context of their complex and diverse personal and environmental needs. However, exemplary for Germany, the Health Literacy Survey-GER 2 [52] showed that people with chronic disease had low navigational health literacy, while they were particularly dependent on information for navigating the health care system. Since the level of health literacy depends not only on individual prerequisites but also on societal and ethnic conditions, concepts to promote health literacy by primary care providers are essential for an equal access to health information. Therefore, the design of health literacy strategies should include both individual capacity building and environmental conditions at the system level [5]. The example of one of the included studies [47] illustrates the need for such multidimensionally, culturally sensitive approaches and for trained health professionals. Smylie et al. reported on the interconnection between health literacy among First Nations people in Canada and colonial policies. Their preliminary researches in a sample of First Nations people with cardiovascular diseases presented lower reported health literacy levels compared to the total population in Canada [53]. The study results showed an improvement in medication knowledge among the patients after educational sessions delivered by a trained nurse [47]. These results verified that building awareness among health professionals of people’s diverse needs is a prerequisite. There is an urgent need to develop system-level strategies that address these dimensions. Research showed that trained nurses with expanded competencies were appropriate multiplicators to provide health literacy promotion and comprehensive information transfer with great achievement in patient satisfaction, compared to doctors [54]. Due to their expanded field of action, community health nurses are suitable to strengthen health literacy and reduce existing inequalities. As a first point of contact, they offer low-threshold and trustworthy access to health care in the neighborhood and for communities. However, community health nurses are still an underappreciated profession in this field. Research on health literacy and advanced nurse-led intervention for Germany is especially scarce [55]. Therefore, this scoping review provides an initial survey of the existing research material on this topic. Community nurse-led approaches to promote health literacy in patients with chronic diseases in primary care practice are presented.

The results showed that there is a large amount of research on health literacy among people with chronic diseases in the context of primary healthcare. Despite the wide range of approaches and methods for health professionals to promote health literacy overall, there is a lack of in-depth analyses. At present, existing studies predominantly focus on the perspective of the entire professional nursing spectrum. However, research regarding the specific requirements of the role of community health nurses is still scarce and poorly differentiated. Studies that addressed the specific needs of community health care or that examine community nurse-led concepts were rare. 

Even though the results of the literature review showed that the transfer of scientific knowledge into practical recommendations for the entire nursing profession spectrum was largely successful. The sources for nurses to acquire methods that address the literacy skills of chronically ill patients are available, but they lack specific concepts for community health nursing. Yet, the demands of practice remain prevalent due to the diversity of the communities (and their needs) and the wide range of responsibilities and peculiarities of CHNs in primary healthcare. The need for CHN-led interventions and their evaluation is implicit. The nine included studies highlighted the positive impact of health literacy strategies implemented by community/advanced nurses on chronic disease management and coping. However, there are not yet many studies. Regarding the comparison of the strategies evaluated, an international comparison seems to be difficult due to the great divergence of the health care system requirements. For this, standardization in future studies is needed in order to improve the comparability of results. 

In the context of considerations on the implementation of CHN in the German health care system, the potentials of advanced nursing competencies for the promotion of patients’ health literacy recently come into focus [31]. Evidence-based tools and training skills in CHN practice will support the development of the autonomous nursing field in Germany. Here, more research is needed with focus on demands of the primary healthcare and the nurse’s role in health literacy interventions in Germany. The expansion of the nursing field in Germany, including health promotion activities offers a great opportunity to establish multidimensional approaches which particularly promote organizational and navigational health literacy [55]. This scoping review is intended to provide an initial overview from which further research questions in this context can be derived. For example, how a community nurse-led intervention that was evaluated as effective (i.e., teach back method) could be integrated into the current primary care practice to improve patient’s health literacy. Additionally, what criteria are required to anchor these new structures in the existing organization.

### Limitations

There were several limitations to the approach of this review. The evidence reported in the included studies was not analyzed. A systematic review is needed for further evaluation. Nevertheless, the two reviewers independently reviewed all the full texts and conducted additional searches. Furthermore, they discussed the inclusion with respect to the set criteria several times. On the one hand, this procedure enhanced the reliability. On the other hand, it limited the number of results included. This may have resulted in failing to identify more important aspects on how CHNs practice in the community setting. Generalizability may also be limited due to the various characterizations of the role of CHNs. 

## 5. Conclusions

This scoping review provided an excerpt of nurse-led strategies and their importance on chronically ill patients’ health literacy in the primary health care setting. A focus on CHNs’ competence/expertise in the field of health literacy research was highlighted. From our full-text assessment, it can be summarized that educational interventions by advanced nurses are essential components for improving chronic disease selfcare outcomes and health literacy. Especially in primary health care, health literacy is a relevant determinant for the quality of treatments regarding chronic conditions. The reviewed literature underlined positive associations between nurse-led communication strategies and positive outcomes in patients with chronic disease. It is consented that embedding/addressing the patients’ health literacy needs and demands is imperative to improve patient’s adherence, knowledge, understanding and behavior. The evidence also supported the importance of health literate communication as a dimension of nurses’ professional competence. The literature emphasized that nurses are key multipliers when it comes to patients’ access to and use of health information. Therefore, there should be a greater understanding of the importance of educating nursing students. Training nursing students in communication skills supports the achievement of a health literate primary care. Although there is a great variety of tools (grey literature) available for nurses to assess patients’ health literacy and to find suitable material to encourage/support them in daily practice, research into evaluated interventions should be strengthened. Above all, this applies to research and the ongoing discourse in Germany. Furthermore, with regard to healthcare gaps in structurally disadvantaged communities, the lack of population-based concepts to promoting health literacy is unsatisfying. Promoting health literacy in people by primary healthcare organizations and staff has different dimensions, depending on its perspective: The (socio-cultural, spiritual) preconditions, circumstances and needs of communities are diverse and group-oriented approaches are needed to strengthen health literacy. Additionally, health literacy researchers agreed: an unfavorable social environment is associated with low levels of health literacy. In this regard, the community setting is important for achieving health equity. This also applies to the promotion of health literacy in communities. The health professional (e.g., CHN), who is the first one contacted for health problems in the community, is able to reach people directly in their living environment and empower them to access, understand and apply health information. In this regard, more research is needed to explore the potential of community nurse-led approaches in the context of primary care.

## Figures and Tables

**Table 1:** Studies included in the literature review in a brief overview.

**Literature Sources Included in Review (*n* = 9)**				
**STUDIES (*N* = 4)**				
**NO.**	**First Author, Year**	**Participants**	**Concept**	**Context**
	Moura et al., 2019 [43]	Adults with type 2 diabetes mellitus	functional health literacy	Nurse-led sessions in primary health care centre [44]
	Yang et al., 2021 [45]	multimorbid individuals with at least three chronic conditions	Medication literacy	Community nurse-led strategy in Community Health Centers
	Mohsen et al., 2021 [46]	adults with diabetes mellitus and hypertension	Health literacy and medication adherence	Community nurse-led educational intervention in primary care
	Smylie et al., 2018 [47]	First Nations people with cardiovascular event, risk and prescribed medication [48]	health literacy in First Nations people	Indigenous nurse-led sessions in primary care
**BRIEF REPORTS (*N* = 2)**				
	Ballard et al., 2016 [27]	Patients with Cancer	Promoting health literacy	Appropriate methods for ANP
	Schäfer, 2022 [49]	Experts in field	Promoting health literacy	Not specified
**GRAY LITERATURE (*N* = 3)**				
	Ledesma, 2021 [50]	Experts in field	CHN/APN methods on health literacy	Community level
	Purvis, 2021 [51]	Adults with type 2 diabetes mellitus	diabetes related health literacy	Educational intervention by ANP
	German Nurses Association, 2022 [31]	Experts in field	Promoting health literacy	Primary care setting

## Data Availability

The data that were generated during the review will be available from the corresponding author on reasonable request.

## Supplementary Materials

The following supporting information can be downloaded at: https://www.mdpi.com/article/10.3390/nursrep13020072/s1, Figure S1: PRISMA flow diagram, as recommended by the PRISMA recommendations; Table S1: Results from the included studies.
