# Supplementary material for: Health Literacy in Adults with Chronic Diseases in the Context of Community Health Nursing: A Scoping Review
Source: Nurs Rep. 2023 May 24;13(2):823–34. doi: 10.3390/nursrep13020072 (PMC10303065; doi:10.3390/nursrep13020072)
Supplement: Supplementary file 1 [file nursrep-13-00072-s001.zip › Table S1.pdf]

Table S1: Results from the included studies

	First author year, country	Study design	Participants (sample) methods	chronic disease/ Aim	Outcomes Questionnaires	Intervention (I) Setting (S)	Main findings
**A**	Moura et al., 2019, Brasil **[43]**	quasi-experimental study pre-/ post intervention	Patients with type 2 diabetes mellitus (at least six months) from 30-69 years registered in two units of Brazilian Family Health Strategy (Estratégia de Saúde da Família, ESF) ESF[1] (sample: n= 55) **[44]**<br><br>ESF's nurses (not further specified) and researcher (PhD in Nursing)<br><br>Measuring adherence to self-care activities and functional health literacy (FHL) in proband four weeks after receiving a nurse-led intervention<br>->Categorization to FHL levels<br>-> Means calculation of days/week and FHL in numeracy domain	type 2 diabetes mellitus<br><br>Assessment of diabetes self-care and FHL and evaluation of effects of an educational intervention	Questionário de Atividades para Autocuidado com o Diabetes (adapted, validated version of the Summary of Diabetes Self-Care Activities Measure (SDSCA) for Brazil)	**I:** 3 sessions (each one about 1 hour) conducted by a ESF nurse and a researcher in a weekly interval (illustrated posters)<br>1. roundtable conversation focusing on knowledge of the causes/ course of diabetes and complication prevention<br>2. roundtable discussion focusing on diabetes self-care intervention (e.g. healthy diet, risks/ adherence to anti-diabetics). Patients practiced self-care measures.<br>3. conversation on foot care using a case experience (pro/contra points on diabetes foot care). Followed by a practical demonstration by the nurse and researcher on specialized foot care procedures (active part).<br>**S**: primary healthcare, at two units of the Brazilian Family Health Strategy (ESF) in Picos, Piauí in Brazil	Total in sample had a low level of FHL in their daily self-care activities<br><br>63.6% of the sample were female and 59.29 years mean age<br><br>Effect on the adherence in "foot care/ shoe inspection" (3.29 days/week difference between means and situated at the analytical level of FHL)<br><br>No increase in "taking insulin shots as recommended"
**b**	Yang et al, 2021 China **[45]**	study protocol for a multicentred, single-blind, two-arm randomised controlled trial 3 months of follow-up	>60 years old multimorbid persons (at least three chronic conditions) with at least one prescribed pharmaceutial and self managing their medications	38 different diseases (not specified)<br>Testing a six-week community nurse-led medication self-management strategy	PO: medication adherence (5-item Medication Adherence Report Scale)<br>SO: Medication knowledge (PKMUQ)	**I:** Intervention with a patient-centred approach delivered by trained CHN in three face-to-face education interviews (each 30-40 min) and 2 weekly follow-up phone calls (10-20 min).	-/-<br>Not described yet (protocol)

[1] [44] Nunes J. Brazil: The Family Health Strategy. **2015** In: Medcalf A., et al. Health For All: The Journey of Universal Health Coverage. Hyderabad (IN): Orient Blackswan; Chapter 13.

		N= estimated sample size: 120 patients<br>(80% power with a two-sided α of 0.05)	Nonadherence to medications<br><br>Community nurses (CHN) will deliver the intervention<br>CHN: junior college degree or above with at least two years of experience in a community health centre (CHC) and daily practice in chronic disease management<br><br>Patients will be randomly allocated to either intervention group (usual care& intervention) or control group (usual care)	and its effect on the medication adherence<br>in comparison to usual care<br><br>Evaluation of the intervention regarding the improvement of medication self-management capacity (medication knowledge)<br>in comparison to usual care	Medication beliefs (BMQ), Medication social support (MSSS)<br>Medication self-efficacy (SEAMS), Medication skills (self-developed scale), Medication treatment satisfaction (TSQM), Treatment burden (TBQ)<br>Quality of life (EQ-5D-5L), Utilisation of healthcare services<br><br>exploring CHNs role in the medication management	1. will determine barriers, inform on medication by the teach-back method<br>2. promote, motivate and empower patient's knowledge by motivational interviewing and encourage social/ family support<br>3. Motivating by planning coping strategies and communication skills on medication adherence<br>4. experience patient's difficulties in selfmanagement by active listening and a personalised feedback<br>**S:** 3 CHCs in primary care in Changsha, China	
**c**	Mohsen et al. 2021, Egypt **[46]**	Quasi-experimental study	adults 60 years and older with chronic diseases (n=140)<br><br>patients are randomly allocated to intervention (intervention) or<br>and control group (usual care and receiving an educational intervention booklet at end of study)<br><br>intervention developed by the researcher (Community Health Nursing, Faculty of Nursing)	Diabetes mellitus and hypertension<br><br>exploring the effect of a health literacy intervention on the medication adherence<br>.	Interviews (survey tested for validity in a pilot study) on socio-demographic data and medical facts<br>Scale on Medication Adherence and the Scale on Self-Efficacy for Appropriate Medication Use<br>Measurement of blood glucose& blood pressure (once/ month in 12 weeks in both groups)	**I:** three sessions (each 20- 30 minutes) in a group with 2- 5 Participants (each patient received an educational intervention booklet per session)<br>1. informing patients on diabetes mellitus and hypertension.<br>2. promotiong diet/ exercise regimen for both diseases<br>3. information on medication adherence and affecting factors<br>**S:** Family health centre in Dragel village, Menoufia Governorate, Egypt	Knowledge, self-efficacy, medication adherence improved significantly in the intervention group<br>decrease of mean blood glucose and mean systolic blood pressure in the intervention group compared to pre-intervention knowledge and to the control group

**d**	Smylie et al. 2018, Canada **[47]**<br><br>(study protocol, design of intervention **[48]**	single arm pre-post study<br><br>(sub study in an international trial on indigenous health services in NZ, AUS, CAN)	First Nations people at DHAC, 20 years or older with least one Cardiovascular event **or** ≥ 15% risk of CVD over the next 5 years and were prescribed at least two medication (statin, aspirin, ACE inhibitors or beta blockers)<br><br>Indigenous nurse who was trained in health literacy education including Indigenous adult literacy strategies<br><br>HL assessed by the nurse on a tablet application (i.e. customized pill card,)	Cardiovascular event (defined as angina pectoris, myocardial infarction, transient ischaemic attack or stroke)<br><br>partnership with an Indigenous health service to test the effect of a customized, structured health literacy educational program addressing CVD medications.	PO: Changes in mean knowledge of CVD medications between pre- and post-educational sessions<br><br>SO: changes in HL practices<br><br>Patients knowledge of CVD medications and their use,	**I:** three educational sessions [2] booklet, developed for the international trial (contained information on CVD diagnosis, medication use, treatment) Application of an tablet with interactive opportunities (animations of stroke and heart disease pathology) and a client-specific pill card 28 days after the first session The nurse actively encouraged clients to practice asking questions about their disease/medications (at health professionals)<br><br>**S:** primary care, De dwa da dehs nye>s Health Access Centre (DHAC) in Hamilton and Brantford, Ontario, Canada.	effects on medication knowledge after all education sessions compared to baseline<br><br>about 90% of participants were able to use the medication booklet and about 50% the pill card<br><br>positive impact in patients on answering questions about their medications<br><br>increased effort on communication with health professionals was not significant
**e**	Ballard et al. 2016, U.S.A. **[27]**	Brief report on practical recommendations (by a case example)	Oncology Nurse's Role in Health Literacy in patients with Cancer (Advanced Nursing Practitioners – ANP)	Cancer-diagnosis<br><br>Recommendation of appropriate methods for ANPs to promote health literacy in patients diagnosed with cancer	-/-	1. Inform patient in plain language on the major objectives and repeat key messages<br>2. Handout written information material limited to three objectives<br>3. Both these strategies followed up by the teach-back method	-/-
**f**	Schäfer 2022, Austria **[49]**	Brief report on promoting health literacy in chronic	A framework is needed to define competencies in clinical/ community settings.	Example of nursing health counseling (nursing core competencies) in	-/-	-/-	appropriate educational offerings (counseling and training) increase

[2] [48] Details reported in:48: Crengle S., Smylie J. Kelaher M., Lambert M., Reid S., Luke J., Anderson I., Hindmarsh JH& Harwood M. Cardiovascular disease medication health literacy among Indigenous peoples: design and protocol of an intervention trial in Indigenous primary care service. **2014** MC Public Health, 14, 714.

	diseases as a task of ANP	Establishment of ANP/CHN is needed in Austria's healthcare as the are key players to promote health literacy by patient education and preventive care planning in patients and population	Austria to promote health literacy (e.g. theory- and concept-guided conversation strategies and conducting training sessions)			health literacy („health coaches")
**Gray Literature (Thesis, project, brochure)**						
**g** Ledesma, 2021, Austria **[50]**	Master thesis<br><br>Qualitative study (Sample: n=9, focus group and experts interviews)	Experts in education, health care and from research projects from Austria, Sweden, Germany and with several years of professional experience	Not specified<br><br>examines how an advanced counseling nurse can help improve health literacy at the community level	Six semi-structured guided expert interviews and one focus group with n=3 experts<br><br>Qualitative content analysis according to Mayring	Explicit CHN/ACN methods on health literacy mentioned in the interviews: (long-term and brief) coaching, (peer-to-peer) counseling, education, evaluation, systemic counseling, reflection, case management, network management participatory concepts<br><br>CHN can effectively operate in the living environment of the patients and identify needs in the community (a new legal framework for CHN is required)<br><br>bachelor/master degree can contribute to this expansion of competencies	CHN/ACN competencies in the primary setting: Communication has to be appropriate to patient's needs<br><br>to be able to provide information in a situation-flexible manner<br><br>information material should be used<br><br>evaluation of practiced strategies is recommended<br><br>networking activity is important
**h** Purvis, 2021 United States **[51]**	A Doctor of Nursing Practice Project	Adults from 18 years with type 2 diabetes mellitus (sample: n=7)<br><br>quality improvement project to evaluate self-management	type 2 diabetes mellitus educating patients in self-management of their diabetes to	After attending six educational classes, a survey was used to evaluate if an increase in confidence in	**I:** six classes (each 1 hour)<br>1. information and discussion on diabetes symptoms and therapy<br>2. information on heart health and preventive stategies /	evidence that participants increased confidence

		education session for patients with diabetes in a rural community where no self management program on diabetes is available<br><br>Intervention was offered by an Advanced Nurse Practitioner	increase their health literacy and to promote heart health, kidney health, nutrition and exercise and to increase their self-confidence	managing their chronic condition occurred.<br><br>Pre-/ post survey<br><br>Assessment of confidence level using a Likert scale from Self-Management Resource Center	lifestyle factors. Communication skills with healthcare providers<br>3. education on diabetes and association to kidney disease.<br>4. education on safely taking diabetes medication.Skills to communicate with primary provider<br>5. Information on healthy food. (supported by MyPlate image)<br>6. Example on an individual's daily exercise<br><br>**S:** Rural health centre in Defuniak Springs,Rural Walton County, Florida, United States	sample size of n= 7 may limit the generalizability of the results.
**9** German Nurses Association (DBfK) 2022, Germany **[31]**	Brochure on Community Health Nursing tasks and practice	Empower/ strengthen health literacy by community nurse-led approaches	-/-	-/-	Promote patient's and relatives health literacy by (individual) information and consultation, by planning events and training programmes and by providing educational materials<br>Accompaniment of quality-related community and urban development processes to improve health literacy in communities	Recommendations on interventions
