# Supplementary figures and images for: Health Literacy in Adults with Chronic Diseases in the Context of Community Health Nursing: A Scoping Review
Source: Nurs Rep. 2023 May 24;13(2):823–34. doi: 10.3390/nursrep13020072 (PMC10303065; doi:10.3390/nursrep13020072)

**Figure S1: PRISMA Flowchart**

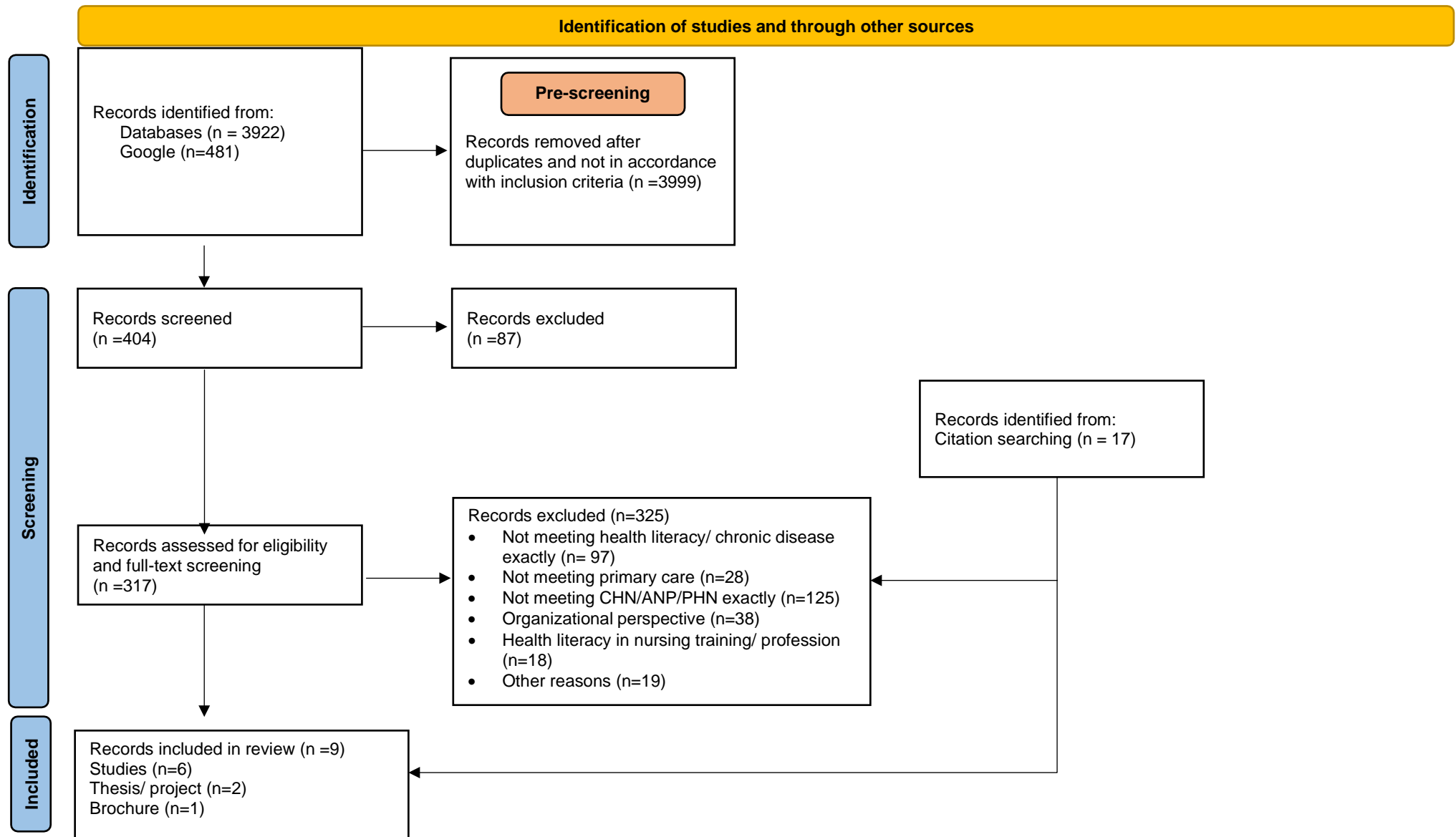

Supplement: Supplementary file 1 [file nursrep-13-00072-s001.zip › Figure S1.pdf]
